# Molecular Fingerprints to Identify *Candida* Species

**DOI:** 10.1155/2013/923742

**Published:** 2013-06-17

**Authors:** Claudia Spampinato, Darío Leonardi

**Affiliations:** ^1^Departamento de Química Biológica, Facultad de Ciencias Bioquímicas y Farmacéuticas, Universidad Nacional de Rosario (UNR), Suipacha 531, 2000 Rosario, Argentina; ^2^Centro de Estudios Fotosintéticos y Bioquímicos (CEFOBI, UNR-CONICET), Suipacha 531, 2000 Rosario, Argentina; ^3^Departamento de Tecnología Farmacéutica, Facultad de Ciencias Bioquímicas y Farmacéuticas, Universidad Nacional de Rosario (UNR), Suipacha 531, 2000 Rosario, Argentina; ^4^Instituto de Química Rosario (IQUIR, UNR-CONICET), Suipacha 531, 2000 Rosario, Argentina

## Abstract

A wide range of molecular techniques have been developed for genotyping *Candida* species. Among them, multilocus sequence typing (MLST) and microsatellite length polymorphisms (MLP) analysis have recently emerged. MLST relies on DNA sequences of internal regions of various independent housekeeping genes, while MLP identifies microsatellite instability. Both methods generate unambiguous and highly reproducible data. Here, we review the results achieved by using these two techniques and also provide a brief overview of a new method based on high-resolution DNA melting (HRM). This method identifies sequence differences by subtle deviations in sample melting profiles in the presence of saturating fluorescent DNA binding dyes.

## 1. Introduction

*Candida* species are opportunistic pathogens which can cause diseases ranging from mucosal infections to systemic mycoses depending on the vulnerability of the host. The major pathogen worldwide is *Candida albicans* [1, 2]. This fungus is detected in the body microbiota of healthy humans [3] and accounts for 75% of the organisms residing in the oral cavity [4]. It is diploid and has a largely clonal mode of reproduction. However, it can undergo considerable genetic variability either by gene regulation and/or genetic changes including chromosomal alterations, mutations, and loss of heterozygosity (LOH). In fact, LOH events lead to *MTL* homozygosis [5], azole resistance [6–8] and microevolution during infection [9–11], passage through a mammalian host [12], or *in vitro* exposure to physiologically relevant stresses [13].

Non-albicans *Candida* species such as *Candida glabrata*, *Candida parapsilosis*, *Candida tropicalis*, *Candida krusei*, and *Candida dubliniensis* are also found with increasing frequency [14–17]. *C. glabrata* has been reported to be the second etiologic agent, after *C. albicans*, of superficial and invasive candidiasis in adults in the United States [18, 19], whereas, in Europe and Latin America, *C. parapsilosis* is the specie responsible for approximately 45% of all cases of candidemia [14, 20].

The ability to discriminate *Candida* isolates at the molecular level is crucial to better understand the spread of these species, particularly in hospitals and to assist in an early diagnosis and initiation of the appropriate antifungal therapy as these organisms show a range of susceptibilities to existing antifungal drugs. *C. albicans*, *C. parapsilosis*, and *C. tropicalis* remain susceptible to polyenes, azoles, and echinocandins [21]. However, *C. glabrata* and *C. krusei* show reduced triazole susceptibility [22, 23]. In addition, the majority of clade 1 isolates of* C. albicans* are less susceptible to flucytosine [24]. The faster and more accurate the species and strains can be identified, the greater the impact in the patient clinical response is. Several methods, such as pulsed-field gel electrophoresis, restriction enzyme analysis, Southern-blot assays, random amplified polymorphic DNA, and amplified fragment length polymorphism, were used to track differences among *Candida* isolates [25, 26]. However, these approaches have limitations such as time consuming, use of radioactive elements, poor reproducibility, and/or discriminatory power [25, 26]. In the present review, we summarize the most exact and/or recent DNA-based techniques developed for a better understanding of the epidemiology of *Candida* species. The availability of the *C. albicans* genome sequence [27–29] facilitated studies in comparative genomics and genome evolution.

## 2. Multilocus Sequence Typing

The multilocus sequence typing (MLST) is based on the analysis of nucleotide sequences of internal regions of various independent housekeeping genes. MLST studies for *C. albicans*, *C. glabrata*, *C. tropicalis*, *C. krusei*, and *C. dubliniensis* have been reported (reviewed in [30]). MLST of *C. albicans* was introduced during the early 2000s [31, 32]. On the basis of a collaborative work, an international consensus set of seven genes for *C. albicans* MLST have been proposed [33]. This gene set includes *AAT1a*, *ACC1*, *ADP1*, *MPIb*, *SYA1*, *VPS13*, and *ZWF1b* (Table 1). *MPIb* has been renamed *PMI1* [34]. Table 1 also shows primers for the amplification and sequencing of the seven gene fragments.

MLST system has proved to be a useful method for epidemiological differentiation of *C. albicans* clinical isolates [31, 32]. Indeed, isolations of *C. albicans* strains recovered from human patients seem to be specific to the patient but not associated with different anatomical sources or hospital origin [9, 10, 35, 36]. MLST studies also revealed a population structure with five major clades of closely related strain types (numbered 1, 2, 3, 4, and 11) plus various minor clades [37]. Clades do not represent cryptic species as genetic exchange between and within clades is limited [38]. Clade 1 is particularly rich in flucytosine-resistant isolates [39, 40]. All clade 1 flucytosine-resistant isolates carry a point mutation (R101C) in the *FUR1* gene which encodes uridine phosphoribosyl transferase [40]. 

A potential weakness of the *C. albicans* international standard gene set is that three of the chromosomes are not represented and two gene pairs are located on the same chromosome (Table 1). In order to include highly informative polymorphisms, a MLST-biased single nucleotide polymorphism (SNP) microarray has been developed [41]. This system which includes 7 loci from the consensus scheme and 12 additional discrete loci located at intervals along the 8 chromosomes may provide a basis for a standardized system.

MLST schemes have been also reported for *C. glabrata* [42]. This typing system is based on fragments of six genes ([42], Table 2). Utilizing this MLST method, several studies have described the population structure of geographically diverse collections of *C. glabrata* isolates [43–45]. Recent MLST analysis of 230 isolates of *C. glabrata* from five populations that differed both geographically and temporally confirmed that the six unlinked loci provide genotypic diversity and differentiation among isolates of this species [46]. MLST studies also revealed that *C. glabrata* strains causing bloodstream infections have similar population structures and fluconazole susceptibilities compared to those normally residing in/on the host [47]. When susceptibility testing of colonizing isolates while receiving azole therapy was studied, MLST revealed the occurrence of resistance development far more frequently in *C. glabrata* than in any other species [48]. This resistance to azole prophylaxis has led to an increased use of echinocandin for primary therapy of *C. glabrata* infections. However, decreased susceptibility to echinocandin drugs can be observed among *C. glabrata* isolates with mutations in the *FKS1* and *FKS2* genes. These genes encode Fks1p and Fks2p subunits of the 1,3-*β*-glucan synthase complex, which synthesizes the principal cell wall component *β*-1,3-glucan, target of echinocandin drugs. In light of this, MLST analysis performed on isolates with *FKS* mutations indicated that the predominant S663P mutation in the *FKS2* gene was not due to the clonal spread of a single resistant phenotype [49].

The MLST system for *C. tropicalis* comprises six housekeeping genes ([52], Table 2). Data indicate that *C. tropicalis* phylogenetically resembles *C. albicans* [53]. Both are diploid organisms, exhibit a predominant clonal mode of reproduction, and support high level of recombination events, which mimic sexual reproduction processes [53]. However, unlike *C. albicans* [35], *C. tropicalis* shows a clonal cluster enriched with isolates with fluconazole resistant or “trailing growth” phenotypes [54]. The term “trailing growth” describes the growth that some isolates exhibit at drug concentrations above the minimum inhibitory concentration (MIC) after 48 h of incubation, although isolates appear fluconazole susceptible after 24 h of incubation. However, Wu et al. [55] reported that *C. tropicalis* isolates were unrelated to the fluconazole resistance pattern, suggesting that the antifungal resistance may develop geographically. Association between the MLST type of each isolate and flucytosine resistance has also been observed [40, 56, 57]. It is interesting that MLST genotypes were only distantly related, thus indicating that flucytosine resistant strains emerged independently in different geographic areas [56].

MLST gene sets for *C. krusei* and *C. dubliniensis* have also been described [50, 51]. Characteristics of the housekeeping loci used for these species are described in Table 2. 

## 3. Microsatellite Length Polymorphisms Analysis

Microsatellite length polymorphisms (MLP) analysis identifies microsatellite instability. Microsatellites, also called simple sequence repeats (SSRs) or short tandem repeats (STRs), are tandem repeat nucleotides comprising 1–6 bp dispersed throughout the genome. These sequences undergo considerable length variations due to DNA polymerase slippage and as a consequence are highly mutagenic [58]. In *Candida* species, this technique has been applied for strain typing [43–45, 59–63], analysis of population structure [64, 65], and epidemiological studies [57, 61, 66–68]. For *C. albicans*, several polymorphic microsatellite loci have been identified (Table 3 and references therein). They were located in the promoter sequence of the elongation factor 3 (*EF3*) [60, 69], in coding regions of extracellular-signal-regulated kinase gene (*ERK1*) [70], downstream of coding sequences of cell division cycle protein (*CDC3*) [59, 60, 71] and imidazole glycerol phosphate dehydratase genes (*HIS3*) [60] and in noncoding regions (CARABEME, CAI, CAIII, CAV, CAVI, and CAVII) [44, 66, 72]. These markers were used alone or in combination. The best discriminatory powers (DPs) obtained were 0.998 for CAI, CAIII, and CAVI [44] and 0.999 for *EF3*, CAREBEME, *CDC3*, *HIS3*, *KRE6*, *LOC4* (*MRE11*), *ZNF1*, CAI, CAIII, CAV, and CAVII [73]. The DP estimates the method ability to differentiate between two unrelated strains. A high DP value (close to 1) indicates that the typing method is able to distinguish each member of a strain population from all other members of that population [74]. It is noteworthy to mention that CA markers were specific for *C. albicans* [44, 66]. In fact, CA microsatellites were named after *C. albicans* and numbered according to the order of the analysis [44]. These markers are highly polymorphic since they are located outside known coding regions, thus being under inconsequential selective pressures. Recently, an allelic *CDC3* ladder has been developed for interlaboratory comparison of *C. albicans* genotyping data [75]. This ladder proved to be important as an internal standard for a correct allele assignment. 

Genotyping systems based on SSR markers have been also described for *C. glabrata*. In 2005, Foulet et al. [67] adopted three polymorphic microsatellite markers located upstream of the mitochondrial RNase P precursor (*RPM2*), metallothionein 1 (*MTI*), and *δ*5,6-sterol desaturase (*ERG3*) genes to generate a rapid strain typing method with a DP of 0.84. These markers were specific for *C. glabrata* isolates. Addition of three new microsatellite markers (GLM4, GLM5, and GLM6) generated a typing system with a DP value of 0.941 [76]. However, by combining only 4 microsatellite markers (*MTI*, *ERG3*, GLM4, and GLM5), authors achieved a DP value of 0.949. A different set of six different microsatellite markers located in noncoding regions (Cg4, Cg5, and Cg6) and in coding regions (Cg7, Cg10, and Cg11) have been described [68], although the highest DP value, 0.902, was reached by using a combination of only four markers (Cg4, Cg5, Cg6, and Cg10). Another research group adopted eight polymorphic microsatellite markers distributed among different chromosomes [77]. This method has a DP value of 0.97, making it suitable for tracing strains. Studies using this system indicate that *C. glabrata* is a persistent colonizer of the human tract, where it appears to undergo microevolution [78]. 

A highly polymorphic CKTNR locus for molecular strain typing of *C. krusei* has been identified [43]. Such locus consists of CAA repeats interspersed with CAG and CAT trinucleotides. Analysis of the CKTNR allele distribution suggested that the reproductive mode of *C. krusei* is mainly clonal [43].

MLP analysis also proved to be a reproducible method for molecular genotyping of *C. parapsilosis* [79]. Seven polymorphic loci containing dinucleotide repeats, most of them located in noncoding regions, were analyzed. The DP calculated for such loci was 0.971. These microsatellites were not amplified with DNA from single representatives of related species, *Candida orthopsilosis* and *Candida metapsilosis* [79]. Recently, another research group conducted *C. parapsilosis* typing studies using one of the previously reported marker (locus B, [79]) and three additional new microsatellite loci located outside known coding regions [80]. This multilocus analysis resulted in a DP of 0.99. These markers were also specific for the molecular typing of *C. parapsilosis* since no amplification products were obtained with DNA of *C. orthopsilosis* and *C. metapsilosis*.

## 4. High-Resolution DNA Melting

**Figure 1:**
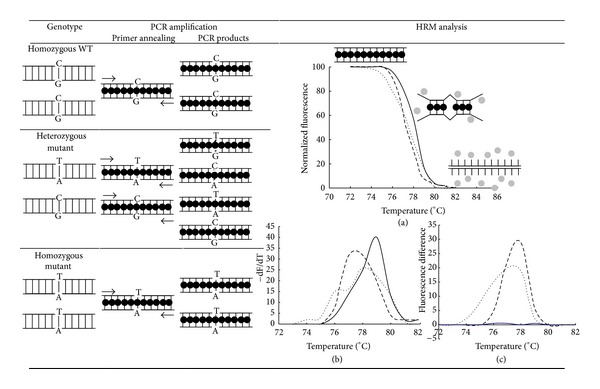
Schematic representation of HRM analysis for SNPs genotyping. Arrows indicate the positions of the primers for allele amplification of a region harboring a SNP. The DNA fluorophore has a bright fluorescence when intercalated to double-stranded DNA (black circle) and low fluorescence in the unbound state (gray circles). Mispaired nucleotides are shown as diagonally broken lines. PCR products from homozygous wild type (solid lines), heterozygous mutant (dotted lines), and homozygous mutant (dashed lines) were analyzed by normalized melting curves (a), derivate melting curves (b), and difference plots (c).

High-resolution DNA melting (HRM) is a novel technique for SNPs genotyping and for the identification of new genetic variants in real time (Figure 1). First, a PCR method is used to amplify specific DNA polymorphic regions in the presence of saturating DNA fluorophores [81]. The dye does not interact with single-stranded DNA but binds to double-stranded DNA, resulting in a bright structure. After PCR amplification, at the beginning of the HRM analysis, the fluorescence is high. As DNA samples are heated up, the double-stranded DNA dissociates releasing the dye which leads to a decrease in the fluorescence intensity (Figure 1(a)). The observed melting temperature (*T*_m_) and the shape of the melt curve are characteristics of the specific sequence of the fragment (primarily the GC content and the length). Data can also easily be interpreted by derivative melting curves (Figure 1(b)) and by plotting the fluorescence difference between a sample and a selected control at each temperature (Figure 1(c)) [81]. Some recent studies used HRM to differentiate clinical *Candida* species [82–84]. HRM has been proven to be a sensitive, reproducible, and inexpensive tool for a clinical laboratory but exhibits low DP values. DP for *CDC3*, *EF3*, and *HIS3* markers was 0.77 [84]. However, HRM can be used along other genotyping methods to increase the resolving power. In fact, the combination of HRM with MLP and SNaPshot minisequencing of the *CDC3* locus provided a DP value of 0.88 [83].

## 5. Conclusions

The development of DNA sequence-based technologies led to a great progress in understanding the epidemiology of clinical isolates of *Candida* species. Both MLST and MLP analysis offer a number of technical advantages over conventional typing methods including extremely high DP values and reproducibility, ease of use, and rapid reliable data. The selection of the technique depends on the purpose of the study, the accessibility of genotypic strains archives, the time available to complete the analysis, and the cost. MLST remains the most reliable method for the assessment of population structure, diversity, and dynamics among *C. albicans*, whereas MLP analysis is most suitable for a rapid and less expensive study of a limited number of isolates.

## Figures and Tables

**Table 1:** International consensus gene set used for *C. albicans* MLST analysis.

Locus	Chromosome	Gene product	Primers	Sequenced fragment size (bp)
Ca*AAT1a *	2	Aspartate aminotransferase	F: ACTCAAGCTAGATTTTTGGC	349
			R: CAGCAACATGATTAGCCC	
Ca*ACC1 *	R	Acetyl-coenzyme A carboxylase	F: GCAAGAGAAATTTTAATTCAATG	407
			R: TTCATCAACATCATCCAAGTG	
Ca*ADP1 *	1	ATP-dependent permease	F: GAGCCAAGTATGAATGATTTG	443
			R: TTGATCAACAAACCCGATAAT	
Ca*PMIb *	2	Mannose phosphate isomerase	F: ACCAGAAATGGCCATTGC	375
			R: GCAGCCATGCATTCAATTAT	
Ca*SYA1 *	6	Alanyl-RNA synthetase	F: AGAAGAATTGTTGCTGTTACTG	391
			R: GTTACCTTTACCACCAGCTTT	
Ca*VPS13 *	4	Vacuolar protein sorting protein	F: TCGTTGAGAGATATTCGACTT	403
			R: ACGGATGGATCTCCAGTCC	
Ca*ZWF1b *	1	Glucose-6-phosphate dehydrogenase	F: GTTTCATTTGATCCTGAAGC	491
			R: GCCATTGATAAGTACCTGGAT	

F and R indicate forward and reverse primers, respectively.

**Table 2:** Summary of loci used for individual MLST schemes. Data for *C. dubliniensis*, *C. glabrata*, *C. krusei*, and *C. tropicalis* are from McManus et al. [50], Dodgson et al. [42], Jacobsen et al. [51], and Tavanti et al. [52], respectively.

Species	Locus	Gene product	Primers	Sequenced fragment size (bp)	Genotypes/site
*C. dubliniensis *	Cd*AAT1a *	Aspartate aminotransferase	F: ATCAAACTACTAAATTTTTGAC	373	1.25
			R: CGGCAACATGATTAGCCC		
	Cd*ACC1 *	Acetyl-coenzyme A carboxylase	F: GCCAGAGAAATTTTGATCCAATGT	407	1.33
			R: TTCATCAACATCATCCAAGTG		
	Cd*ADP1 *	ATP-dependent permease	F: GAGCCAAGTATGAATGACTTG	443	1.2
			R: TTGATCAACAAACCCGATAAT		
	Cd*PMIb *	Mannose phosphate isomerase	F: ACCAGAAATGGCC	375	3.5
			R: GCAGCCATACATTCAATTAT		
	Cd*RPN2 *	26S proteasome regulatory subunit	F: TTTATGCATGCTGGTACTACTGATG	302	1
			R: TAACCCCATACTCAAAGCAGCAGCCT		
	Cd*SYA1 *	Alanyl-RNA synthetase	F: AGAAGAATAGTTGCTCTTACTG	391	1
			R: GTTGCCCTTACCACCAGCTTT		
	Cd*VPS13 *	Vacuolar protein sorting 13	F: CGTTGAGAGATATTCGACTT	403	1.33
			R: ACGGATCGATCGCCAATCC		
	Cd*ZWF1b *	Glucose-6-phosphate dehydrogenase	F: GTTTCATTTGATCCTGAAGC	491	0.86
			R: GCCATTGATAAGTACCTGGAT		

*C. glabrata *	Cg*FKS *	1,3-*β*-glucan synthase	F: GTCAAATGCCACAACAACAACCT	589	1.27
			R: AGCACTTCAGCAGCGTCTTCAG		
	Cg*LEU2 *	3-Isopropylmalate dehydrogenase	F: TTTCTTGTATCCTCCCATTGTTCA	512	1
			R: ATAGGTAAAGGTGGGTTGTGTTGC		
	Cg*NMT1 *	Myristoyl-coenzyme A, protein N-myristoyltransferase	F: GCCGGTGTGGTGTTGCCTGCTC	607	0.81
			R: CGTTACTGCGGTGCTCGGTGTCG		
	Cg*TRP1 *	Phosphoribosyl-anthranilate isomerase	F: AATTGTTCCAGCGTTTTTGT	419	1.08
			R: GACCAGTCCAGCTCTTTCAC		
	C*gUGP1 *	UTP-glucose-1-phosphate uridylyltransferase	F: TTTCAACACCGACAAGGACACAGA	616	0.75
			R: TCGGACTTCACTAGCAGCAAATCA		
	Cg*URA3 *	Orotidine-5′-phosphate decarboxylase	F: AGCGAATTGTTGAAGTTGGTTGA	602	0.68
			R: AATTCGGTTGTAAGATGATGTTGC		

*C. krusei *	Ck*ADE2 *	Phosphoribosylaminoimidazole carboxylase	F: GTCACTTCTCAGTTTGAAGC	470	2.33
			R: ACACCATCTAAAGTAGAGCC		
	Ck*HIS3 *	Imidazole glycerol phosphate dehydratase	F: GGAGGGGACATATCACTGCC	400	1.75
			R: AATCTTTAATTGCCAAAGCC		
	Ck*LEU2 *	3-Isopropylmalate dehydrogenase	F: CTGTGAGACCAGAACAGGGG	619	1.89
			R: GCAGAGCCACCCAAGTCTCC		
	Ck*LYS2 *	L-Aminoadipate-semialdehyde dehydrogenase	F: ATCTGAGAAGCAGTTGGCGC	441	1.90
			R: AGACTTGTAAGAATTATCCC		
	Ck*NMT1 *	Myristoyl-coenzyme A, protein N-myristoyltransferase	F: CTGATGAAGAAATCACCG	537	2.00
			R: GCTTGATATCATCTTTGTCC		
	Ck*TRP1 *	Phosphoribosyl-anthranilate isomerase	F: AGCTATGTCGAGCAAAGAGG	380	2.00
			R: ACATCAACGCCACAACACCC		

*C. tropicalis *	Ct*ICL1 *	Isocitrate lyase	F: CAACAGATTGGTTGCCATCAGAGC	447	0.71
			R: CGAAGTCATCAACAGCCAAAGCAG		
	Ct*MDR1 *	Multidrug resistance protein	F: TGTTGGCATTCACCCTTCCT	425	1.67
			R: TGGAGCACCAAACAATGGGA		
	Ct*SAPT2 *	Secreted aspartic protease 2	F: CAACGATCGTGGTGCTG	525	0.51
			R: CACTGGTAGCTGAAGGAG		
	Ct*SAPT4 *	Secreted aspartic protease 4	F: TGCTTCTCCTACAACTTCACCTCC	390	0.90
			R: ATTCCCATGACTCCCTGAGCAACA		
	Ct*XYR1 *	D-xylose reductase I or II	F: AGTTGGTTTCGGATGTTG	370	3.00
			R: TCGTAAATCAAAGCACCAGT		
	Ct*ZWF1 *	Glucose-6-phosphate dehydrogenase	F: GGTGCTTCAGGAGATTTAGC	520	0.94
			R: ACCTTCAGTACCAAAAGCTTC		

F and R indicate forward and reverse primers, respectively. Genotypes/site indicate the ratio of genotypes to SNPs.

**Table 3:** Description of SSRs used for MLPs analysis of *C. albicans. *

	Locus	Ch	Gene product	Repeat motif	Location	DP	Primers	References
1	*EF3 *	5	Elongation factor 3	(TTC)_*n*_ (TTTC)_*n*_	Upstream region	0.88_(1)_ 0.97_(1+3+4)_ 0.999_(ALL except 2+12)_	F: TTTCCTCTTCCTTTCATATAGAA R: GGATTCACTAGCAGCAGACA	[59, 60, 64, 69, 73]
2	*ERK1 *	4	Extracellular-signal-regulated kinase	(CAGGCT)_*n*_(CAAGCT)_*n*_ - -(CAA)_*n*_- -(GCCGCA)_*n*_ - -(CTT)_*n*_	Coding region	nr	F: CGACCACGTCATCAATAGAAATCG R: CGTTGAATGAAACTTGACGAGGGG	[64, 70]
3	CARE-BEME	6	nc	(GAA)_*n*_	Noncoding region	0.999_(ALL except 2+12)_	F: GAATCATGAAACAGAAACTG R: TGGGTGAAGGATAATCTGCA	[72, 73]
4	*CDC3 *	1	Cell division cycle protein	(AGTA)_*n*_	Downstream region	0.97_(1+3+4)_ 0.999_(ALL except 2+12)_	F: CAGATGATTTTTTGTATGAGAAGAA R: CAGTCACAAGATTAAAATGTTCAAG	[60, 73]
5	*HIS3 *	2	Imidazole glycerol phosphate dehydratase	(ATTT)_*n*_	Downstream region	0.97_(1+3+4)_ 0.999_(ALL except 2+12)_	F: TGGCAAAAATGATATTCCAA R: TACACTATGCCCCAAACACA	[60, 73]
6	*KRE6 *	3	*β*-1,6-Glucan synthesis	(AAT)_*n*_	Coding region	0.999_(ALL except 2+12)_	F: CAAGCTTATAGTGGCTACTA R: CCAACACTGATACATCTCG	[64, 73]
7	*LOC4* (*MRE11*)	7	Double-strand break repair protein	(GAA)_*n*_	Coding region	0.999_(ALL except 2+12)_	F: GTAATGATTACGGCAATGAC R: AGAACGACGTGTACTATTGG	[64, 73]
8	*ZNF1 *	4	Zinc finger transcription factor	(CAA)_*n*_	Coding region	0.999_(ALL except 2+12)_	F: CCATTACAGCTGAACCAGCGAGGG R: CGCTAGGTAACCTACAGATTGTGGC	[64, 73]
9	CAI	4	nc	(CAA)_*n*_- -(CAA)_*n*_	Noncoding region	0.967_(9)_ 0.998_(9+10+12)_ 0.999_(ALL except 2+12)_	F: ATGCCATTGAGTGGAATTGG R: AGTGGCTTGTGTTGGGTTTT	[44, 66, 73]
10	CAIII	5	nc	(GAA)_*n*_	Noncoding region	0.853_(10)_ 0.998_(9+10+12)_ 0.999_(ALL except 2+12)_	F: TTGGAATCACTTCACCAGGA R: TTTCCGTGGCATCAGTATCA	[44, 73]
11	CAV	3	nc	(ATT)_*n*_	Noncoding region	0.853_(11)_ 0.999_(ALL except 2+12)_	F: TGCCAAATCTTGAGATACAAGTG R: CTTGCTTCTCTTGCTTTAAATTG	[44, 73]
12	CAVI	2	nc	(TAAA)_*n*_	Noncoding region	0.853_(12)_ 0.998_(9+10+12)_	F: ACAATTAAAGAAATGGATTTTAGTCAG R: TGCTGGTGCTGCTGGTATTA	[44]
13	CAVII	1	nc	(CAAAT)_*n*_	Noncoding region	0.670_(13)_ 0.999_(ALL except 2+12)_	F: GGGGATAGAAATGGCATCAA R: TGTGAAACAATTCTCTCCTTGC	[44, 73]

Discriminatory power (DP) based on one or various loci is indicated in brackets according to the row number. Dashed line indicates various nucleotides that separate different microsatellites in the sequence. Ch: chromosome; nc: not corresponding; nr: not reported.
